# Shotgun Lipidomics by Sequential Precursor Ion Fragmentation on a Hybrid Quadrupole Time-of-Flight Mass Spectrometer

**DOI:** 10.3390/metabo2010195

**Published:** 2012-02-20

**Authors:** Brigitte Simons, Dimple Kauhanen, Tuulia Sylvänne, Kirill Tarasov, Eva Duchoslav, Kim Ekroos

**Affiliations:** 1 AB SCIEX, 71 Four Valley Dr. Concord, ON L4K4V8, Canada; Emails: brigitte.simons@absciex.com (B.S.); eva.duchoslav@absciex.com (E.D.); 2 Zora Biosciences, Biologinkuja 1, Espoo, FI-2150, Finland; Emails: dimple.kauhanen@zora.fi (D.K.); tuulia.sylvanne@zora.fi (T.S.); kirill.tarasov@zora.fi (K.T.)

**Keywords:** MS/MS^ALL^, lipidomics, lipid, QTOF, mass spectrometry

## Abstract

Shotgun lipidomics has evolved into a myriad of multi-dimensional strategies for molecular lipid characterization, including bioinformatics tools for mass spectrum interpretation and quantitative measurements to study systems-lipidomics in complex biological extracts. Taking advantage of spectral mass accuracy, scan speed and sensitivity of improved quadrupole linked time-of-flight mass analyzers, we developed a bias-free global lipid profiling acquisition technique of sequential precursor ion fragmentation called MS/MS^ALL^. This generic information-independent tandem mass spectrometry (MS) technique consists of a Q1 stepped mass isolation window through a set mass range in small increments, fragmenting and recording all product ions and neutral losses. Through the accurate MS and MS/MS information, the molecular lipid species are resolved, including distinction of isobaric and isomeric species, and composed into more precise lipidomic outputs. The method demonstrates good reproducibility and at least 3 orders of dynamic quantification range for isomeric ceramides in human plasma. More than 400 molecular lipids in human plasma were uncovered and quantified in less than 12 min, including acquisitions in both positive and negative polarity modes. We anticipate that the performance of sequential precursor ion fragmentation both in quality and throughput will lead to the uncovering of new avenues throughout the biomedical research community, enhance biomarker discovery and provide novel information target discovery programs as it will prospectively shed new insight into affected metabolic and signaling pathways.

## 1. Introduction

The unprecedented advances in mass spectrometry (MS), combined with the need for comprehensive lipid measurements from the research and medical communities, have led to the rapid evolution of lipidomics. The prime focus of lipidomics is to perform systems-level analysis of lipid species, and uncover their abundance, biological activities, subcellular localization and tissue distribution [1]. Today, lipidomics has proven to be the most essential discipline for the discovery of disease-related metabolic dysfunctions and promoting the discovery of disease biomarkers and new drug targets.

The lipidome of mammalian plasma is believed to contain thousands of lipid entities that structurally and chemically regulate cell membranes, store energy, or become precursors to bioactive metabolites [2]. Lipids primarily reside in cellular membranes and their bioactive outputs are determined collectively by the individual molecular lipids present [3]. The molecular lipid structures, their local concentrations and spatial distributions will therefore define the physiological response. Indeed, several studies have clearly demonstrated and highlighted the importance and specificity of detailed molecular lipid structures in determining bio-functionality. For instance, Menuz and colleagues have shown that C24- to C26-carbon ceramides mediated the death of a *C. elegans* mutant that failed to resist asphyxia, whereas ceramides with shorter chains had the opposite effect [4]. We have recently showed that the C24-carbon ceramide induced ER stress, whereas the shorter chain (C20-22 and C16) ceramides had no effect in HL-1 cardiomyocyte cells [5]. Based on these above-mentioned studies, it can be anticipated that a defect in underlying lipid regulation can lead to deleterious effects on the organism and assist in the pathophysiology of diseases. Therefore, the analysis of molecular lipid species is essential, since these biological entities have potential impact in multiple research fields ranging from basic science to clinical diagnostics.

Tandem mass spectrometry strategies have proven to be well suited for achieving detailed characterization of lipid molecular species. Recently, Quehenberger and colleagues described an extensive profiling of mammalian lipids in plasma and quantitative analysis of more than 500 lipid species [6]. We have established a high-throughput workflow to routinely accomplish similar analyses, however, with the essential exception that all lipids are identified and quantified as molecular species [7]. Lipid classes such as glycerophospholids, glycerolipids, glycosphingolipids and sterol lipids can be identified by distinguishing their characteristic headgroup ions, long-chain bases, fatty acid acyl ions and corresponding neutral losses. Classically, these types of ions have been used in precursor ion scanning (PIS), neutral loss scanning (NLS) and multiple reaction monitoring (MRM) experiments, primarily carried out on triple quadrupole (QqQ) instruments due to the high sensitivity, favorable quantitative capabilities, acquisition speed and selectivity of this technology [8,9,10,11]. These analysis modes have successfully been applied to both, coupled with either direct infusion, e.g., shotgun lipidomics, or liquid chromatography (LC) modes for multiplexing several lipid-class targeted experiments, simultaneously including internal standards and based on total lipid extracts [12,13,14,15,16]. However, the drawback of such analyses is that they require the selection of characteristic ions in advance and are highly targeted. Currently, it is neither experimentally nor technologically feasible to perform untargeted experiments in this way, due to the vast number of theoretical ions that would need to be pre-selected for the analysis. Instead, shotgun or LC-based information-dependent acquisition (IDA) experiments have proven more effective for untargeted lipid identification. IDA experiments are typically based on prioritizing a list of candidate precursor ions in real time, based on survey MS data and user-defined information dependent criteria, such as peak intensity, and selecting these ions for MS/MS. However, some of the challenges of this approach include: lack of reproducibility, unrestrained MS/MS data quality and laborious analyses.

Here, we present an alternative approach to the IDA technique, utilizing the latest features of the hybrid quadrupole time-of-flight (QTOF) technology, employing an approach for sequential precursor ion fragmentation (MS/MS^ALL^). MS/MS^ALL^ is a simple direct infusion information-independent acquisition technique of stepping through a pre-defined mass range in small increments and effectively fragmenting everything within the set mass range. In the absence of IDA prioritization, all precursors are selected in the Q1 quadrupole at unit-based resolution in a step-wise fashion to completely cover the entire mass range of interest. Collision-induced dissociation (CID) is carried out in Q2 at high speed, while collecting more than a thousand of MS/MS spectra covering every precursor in the mass range of each cycle. The MS/MS^ALL^ approach is bias-free and potentially delivers very informative product ion spectra, even in the absence of an MS precursor ion signal. The MS/MS data can further be directly applied towards batch library searching or any spectral MS^2^-level algorithms for a more succinct unambiguous lipid molecular profiling workflow. As highlighted in previous publications describing such workflows using benchtop orbitrap instrumentations [17] and linear ion traps [17,18], sequential precursor ion fragmentation in a single experiment is highly desirable for numerous applications as nothing is missed, all masses are simply fragmented, independent of signal intensity, and data can be mined retrospectively. As the recent QTOF instrumentation can be attributed with the sensitivity of a triple quadrupole system as well as with high resolution throughout, a broad mass range, full coverage MS/MS data, acquired with high mass accuracy (≤2 ppm) offer greater capability towards the identification and confirmation of lipids in complex biological extracts. MS/MS^ALL^ represents a novel scanning technique amenable to the analysis of biological lipid extracts and automated untargeted high throughput molecular lipidomics.

## 2. Results and Discussion

### 2.1. Workflow of the Sequential Precursor Ion Fragmentation

**Figure 1:**
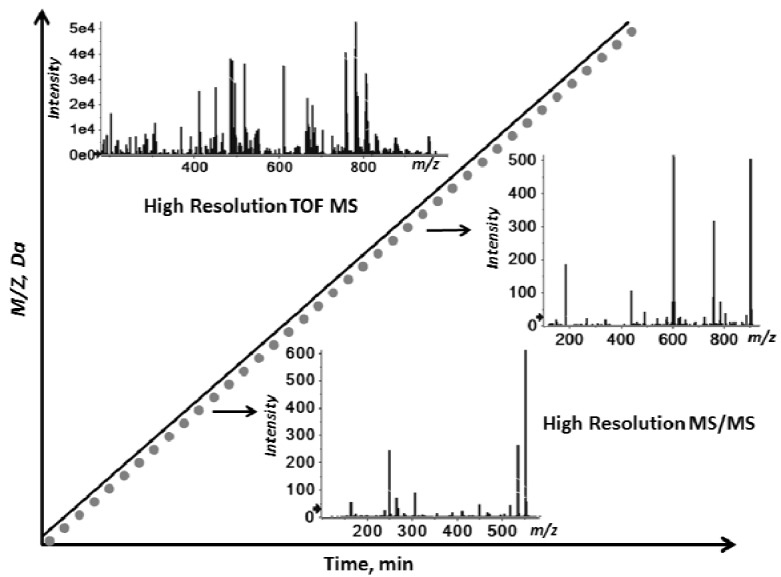
Schematic overview of MS/MS^ALL^. In a set mass range, precursors are sequentially selected in the Q1 quadrupole at unit-based resolution in a step-wise fashion, subjected to fragmentation in the collision cell Q2, and the generated fragment ions are monitored at high resolution by time-of-flight. In addition to the ordered MS/MS, a high resolution survey TOF MS is acquired.

An untargeted, shotgun lipidomics workflow was developed consisting of the ordered acquisition of high resolution, accurate mass time-of-flight detection of all precursor and product ions: an acquisition technique termed MS/MS^ALL^. This LC-free workflow is generic; no extensive method development or optimization was performed. The concept, as illustrated in Figure 1, is to carry out a high resolution TOF MS scan covering a user-defined mass range followed by a series of high resolution MS/MS experiments collected in the order corresponding to each Q1 precursor isolation step (stepped by approximately 1 amu). The collision energy was ramped over 40 eV during each MS/MS step to provide a sweep of fragmentation accumulated for high coverage of precursor-derived fragment products. By taking advantage of the rapid data collection that the current technology offers, *i.e*.,accumulation time of 300 milliseconds, a complete sequential precursor ion fragmentation experiment was accomplished in less than six minutes. Since both positive and negative polarity modes can be acquired in serial experiments from a single sample infusion, an approximately 12 min analysis time is required to collect a complete chemical record of all ions at high resolution for that particular sample.

### 2.2. Overview of the Experimental Setup

**Figure 2:**
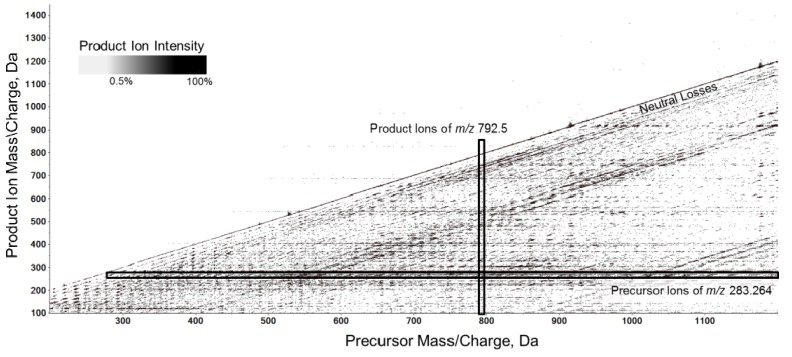
A complete fragmentation map in negative ion mode of all precursor ions from *m/z* 200–1,200 (x-axis), neutral loss (diagonal), and product ions from *m/z* 100–1,500 (y-axis) generated by a single cycle of the MS/MS^ALL^ acquisition.

The potential of the sequential precursor ion fragmentation technique was investigated on total plasma lipid extracts obtained from healthy donors. The plasma samples were introduced to the mass spectrometer using a flow injection setup as described in the experimental section. We acquired a short TOF MS scan from 200*–*1,200 *m/z*, followed by 1,000 individual MS/MS experiments applying approximately 1 amu step size according to the description above. Positive and negative mode acquisitions were acquired back-to-back with appropriate blanks between samples. The carry-over from sample to sample was assessed to be less than 2% (data not shown). Overall quality and information captured in the collected data for one sample were visualized in a three-dimensional contour plot, which depicts a complete set of all precursor ions, product ions and neutral losses from a single cycle in the MS/MS^ALL^ acquisition mode. A contour plot of a negative ion mode acquisition of a plasma total lipid extract is shown in Figure 2. The output assists to identify precursor ions that share specific fragment ion signatures and pinpoint the fragment ions of a particular precursor ion. This way of visualization simplifies the read-out of such a complex data set and assists in the initial step of lipid fingerprinting between samples for the information of interest (analogous to 2D gel electrophoresis for proteomics). Notably, other tools are required for further in-depth analyses of the acquired data, including lipid identification, quantification and statistical analyses.

We further assessed the suitability of the acquired data for determination of complex lipidomes. This included evaluation of the quality, quantification and reproducibility aspects of the chosen methodology. Finally, we evaluated performance and throughput in determining the human plasma lipidome, including comparative analyses attained with other approaches.

### 2.3. High Resolution Accurate MS Improves Lipid Identification but Does Not Reveal Underlying Molecular Species

**Figure 3:**
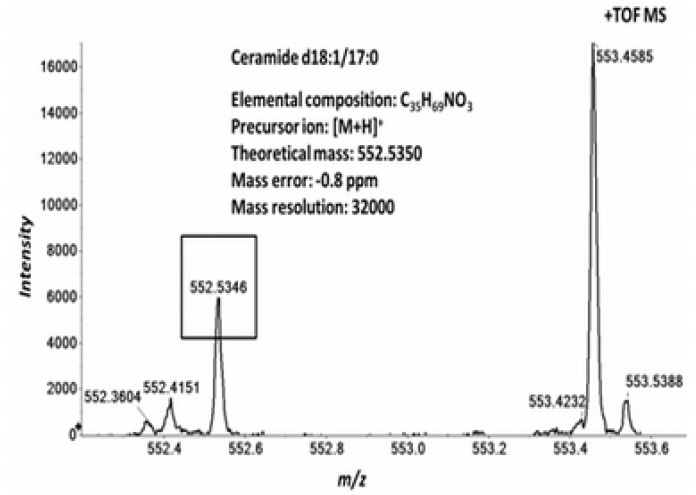
Identification of ceramide d18:1/17:0 by TOF MS. Human plasma total lipid extracts were spiked with the isobaric CER d18:1/17:0 standard and analyzed as described in the Experimental section. Positive ion mode TOF MS reveals the presence of the ceramide as a sum composition, identified by the observed peak at *m/z* 552.5346, corresponding to the protonated complete ceramide molecular ion, [M+H]^+^. The peak was detected at a mass resolution of 32000.

We first investigated the performance of the TOF MS acquisition. We spiked the plasma samples with the ceramide d18:1/17:0 (CER d18:1/17:0) lipid standards prior to lipid extraction [7]. Ceramides are typically recognized as [M+H]^+^ and [M+H−H_2_O]^+^ in positive ion mode [19]. The theoretical mass of the [M+H]^+^ of the CER d18:1/17:0 is 552.5350 Da. In the positive ion mode TOF MS analysis we identified the ceramide species at *m/z* 552.5346 (Figure 3). The measured mass error was 1.0 ppm, demonstrating the high mass accuracy of the TOF and that this analysis alone is sufficient to identify the selected ceramide species. 

**Figure 4:**
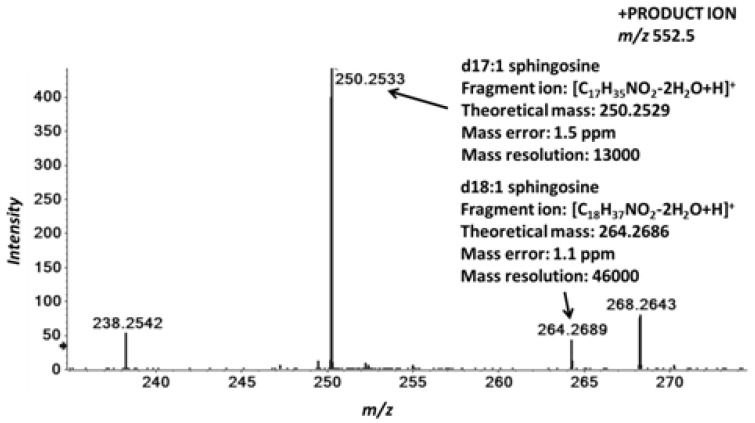
Identification of isobaric ceramides by MS/MS^ALL^. Human plasma total lipid extracts were spiked with the isobaric CER d18:1/17:0 and CER d17:1/18:0 standards and analyzed as described in the Experimental section. Positive ion mode MS/MS of the precursor ion *m/z* 552.5 confirms the identification of the isobaric ceramides CER d18:1/17:0 and CER d17:1/18:0 by the detection of their characteristic fragment ions at *m/z* 250.2533 and *m/z* 264.2689, respectively. The mass range *m/z* 235–275 is shown.

### 2.4. Accurate Mass Product Ion Data Are Required to Confirm Molecular Lipids

Lipidomes comprise molecular lipids of which many are isobaric and isomeric [20,21] (see below). A full MS scan analysis alone, even with the highest mass accuracy, cannot resolve molecular ions having identical mass, rather it yields lipids species as total or sum compositions. Additional information from the underlying lipid-specific characteristic fragment ions is required to aid in determining molecular species. Therefore, to mimic the real-life scenario where numerous isomeric and isobaric species are present in the samples, we added the synthetic isomeric ceramide standard CER d17:1/18:0, in order to determine whether MS/MS^ALL^ is capable of identifying these species in parallel. In the acquired MS/MS spectra of the precursor ion window of *m/z* 552.5–553.5, we could readily identify the characteristic sphingosine-derived fragment ions [19] (Figure 4). The d18:1 and d17:1 sphingosine-derived fragment ions were detected at *m/z* 264.2689 and *m/z* 250.2533, respectively. The mass error calculated against their theoretical masses was shown to be below 1.5 ppm, thus aiding in unambiguous identification of both ceramide molecular species.

### 2.5. Sequential Precursor Ion Fragmentation Delivers Quantitative Data

The sphingosine-derived fragment ions have been commonly used on QqQ instruments for the quantitative analysis of ceramides by PIS [19] and MRM [9,22]. However, the limited mass resolution on QqQ platforms can jeopardize quantification due to interfering ions that have similar masses to the one(s) of interest. This applies to both the shotgun- and the LC-based techniques, despite the chromatographic separation step in the latter method [7].

**Figure 5:**
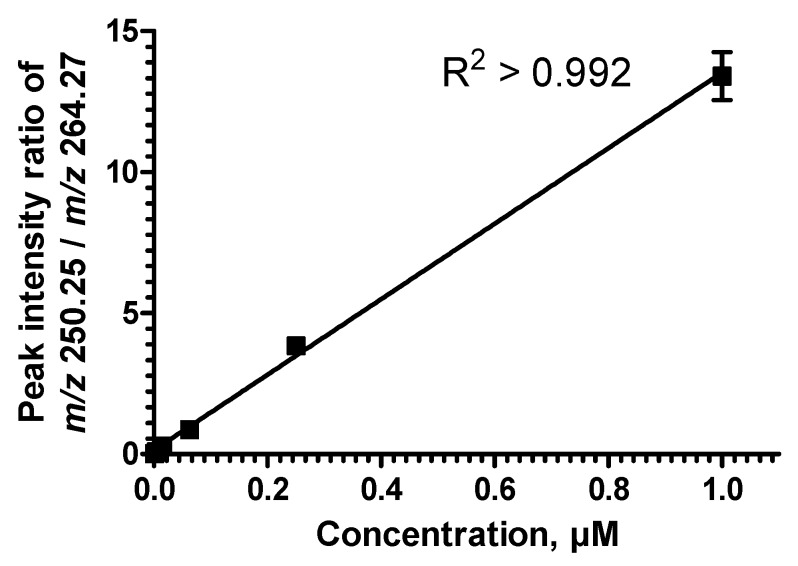
MS/MS^ALL^ is quantitative. Human plasma total lipid extracts spiked with the isobaric ceramide standards (shown in Figure 4) were utilized for evaluating the linear instrument response. The peak intensities of the fragment ions corresponding to d17:1 sphingosine and d18:1 spingosine, respectively, were obtained from the MS/MS^ALL^ acquisitions. Synthetic CER d17:1/17:0 standard was serial diluted (0.001 µM to 1 µM) relative to a constant amount of the synthetic CER d18:1/17:0 standard. The y-axis shows the ratio of the intensities of spectral peaks at the *m/z* 250.25 and 264.27 and the x-axis represents the absolute concentration of CER d18:1/17:0 from three independent calibration data series (six different concentrations). R^2^ linear regression value was 0.992. Error bars indicate standard deviation (n = 3).

Utilizing the accurate mass fragment ions to achieve accurate quantification was demonstrated previously [23]. In order to evaluate the linear response of the MS/MS^ALL^ technique and its dynamic quantification range based on sphingosine-derived fragment ions, we prepared a serial dilution of the CER d17:1/18:0 in human plasma while the concentration of CER d18:1/17:0 was kept constant. We plotted the peak intensity ratio of the *m/z* 250.25 to *m/z* 264.27 against the spiked concentration of the CER d17:1/18:0 standard (Figure 5). A linear calibration line was obtained ranging from 0.001 μM to 1.0 μM, with an R^2^ regression value of 0.992. Based on the calibration line, the quantification limit is in the low nanomolar range for these particular lipid species. These results agree with previous work on hybrid QTOF instrumentations [23]. In addition, the new technology provides improved sensitivity at millisecond accumulation rates almost reaching the level of the latest QqQ technology using PIS of *m/z* 264.3 (data not shown). Performing the same experiments on QqQ technology resulted in very similar calibration line slopes (data not shown). The reason behind the minor differences in the calibration slopes between the two platforms is largely due to differences in system setup and flow rates, e.g., influence of make-up flow *vs.* direct nanoflow infusion [24], or the interference of background ions as discussed above. At this point we did not pursue to investigate this further.

### 2.6. Reproducibility of the Sequential Precursor Ion FragmentationTechnique

The pre-defined MS/MS precursor sequence in each cycle of the MS/MS^ALL^ workflow ensures that a dataset is collected with maximum reproducibility. The reproducibility of the technique was assessed by monitoring response of synthetic lipid standards in a series of three technical replicates of human plasma. The intensities of characteristic fragments of monitored lipids gave a coefficient of variation (CV) in the range of 2–10% in positive ion mode (Table 1); in negative ion mode the CV range was 1–11%. Overall, the MS/MS^ALL^ technique delivered, irrespective of the polarity mode, a median CV of approximately 5%.

### 2.7. MS/MS^ALL^ Accompanied with High Resolution MS Increases the Confidence in Endogenous Lipid Identification

**Figure 6:**
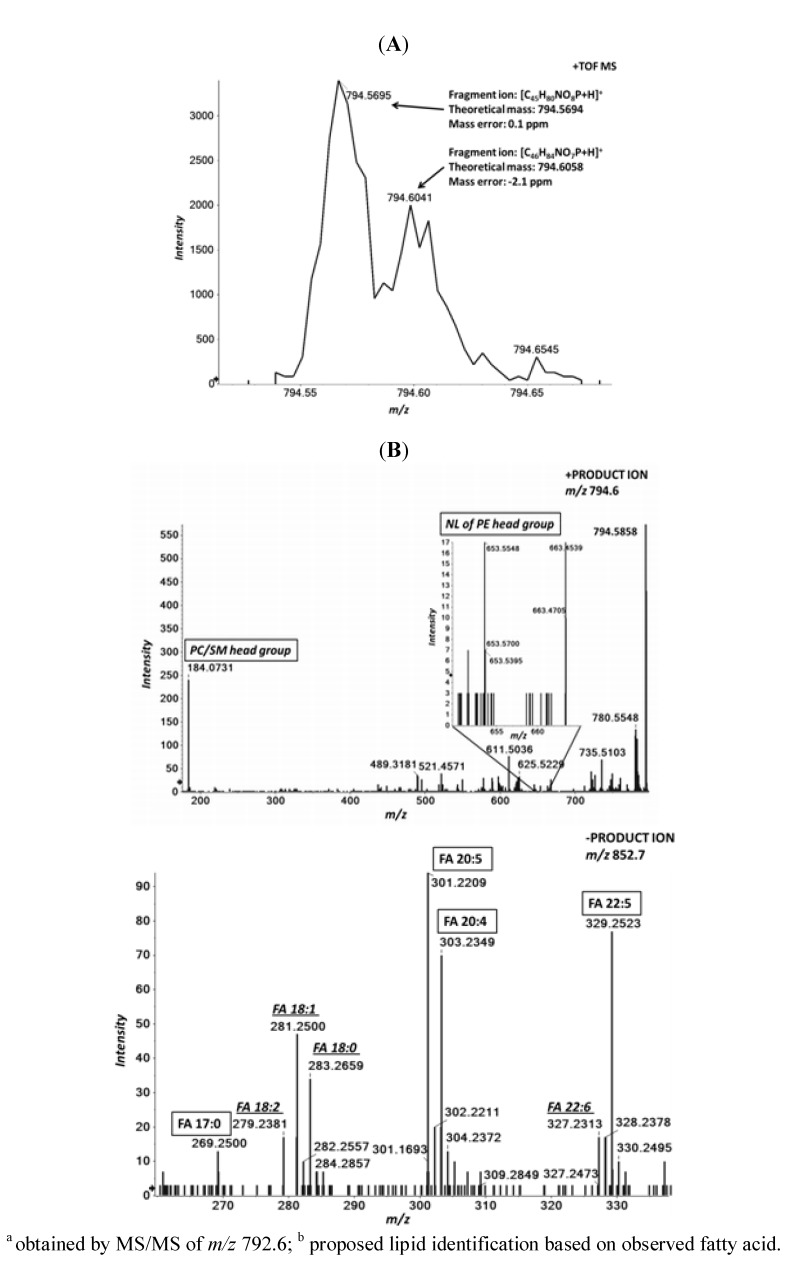
MS/MS^ALL^ expedites identification of isobaric lipids in human plasma. Human plasma total lipid extracts were prepared and analyzed as described in the Experimental section. (**A**) Positive ion mode TOF MS reveals two distinct peaks at *m/z* 794.5695 and at *m/z* 794.6058 suggesting the presence of both PC 37:5 and PC O-38:5, based on their masses; (**B**) Positive ion mode MS/MS of the precursor ion (*m/z* 794.6) confirms the presence of PC, based on the detection of the phosphoryl choline head group (*m/z* at 184.0731), but simultaneously reveals the presence of PE species based on the detection of the mass at *m/z* 653.5548, corresponding to the NL of the PE head group [11]; (**C**) Negative ion mode MS/MS of the precursor ion monitored as an acetate adduct (*m/*z 852.7), detects the underlying acyl anions. Collectively the obtained results propose the presence of PC 17:0/20:5, PC P-18:0/20:4, PC O-18:0/20:5 and PC O-16:0/22:5 (Table 2). The raw MS/MS further suggests the presence of overlapping lipid species based on the detected acyl ions (italic underlined) which partially originate from isotope peaks of lower mass lipids. The MS/MS scan was performed in both polarity modes using collision energy of 50 eV and the data was de-isotoped.

As MS/MS^ALL^ proved to deliver both accurate and quantitative data for spiked lipid species, we proceeded to evaluate its performance on selected endogenous lipid species of human plasma. Distinguishing alkyl- (O) and alkenyl (P) linked phosphatidylcholines (PC) from diacyl PCs have been hampered due to their similar masses and the overlapping characteristic fragment ions generated upon CID. For instance, the mass difference between the total PC 37:5 (total number of carbons:total number of double bonds in attached fatty acids) and total PC-O 38:5 is only about 36 mDa. Based on the resolution of the instrument and the mass accuracy achieved in the ceramide experiments discussed above, a TOF MS acquisition should be sufficient to distinguish between these two isobaric species. Indeed, in positive ion mode two peaks at *m/z* 794.5674 and *m/z* 794.6034, corresponding to PC 37:5 and PC-O 38:5, respectively, were detected (Figure 6A). The mass error for these minor species was less than 3 ppm, even with the signal-to-noise of less than 5 (peak-to-peak). Thus, this indicates that the mass accuracy can be retained even for minor peaks. 

**Table 1:** Reproducibility of the sequential precursor ion fragmentation technique.

Synthetic standard	Measured *m/z*	Peak intensity (cps)			Mean (n = 3)	CV (%)
		Sample A	Sample B	Sample C		
LPC 17:0	184.07	1070	1165	966	1067	9.33
D6-CE 18:0	369.35	810	916	896	874	6.44
PC 17:0/17:0	184.07	12360	12056	12775	12397	2.91
D3-GlcCER d18:1/16:0	264.27	282	302	282	288	4.00
LPC 17:0	269.25	2875	2893	3496	3088	11.45
PC 17:0/17:0	269.25	3559	4211	3821	3864	8.49
PE 17:0/17:0	269.25	8682	8536	8629	8616	0.86
PS 17:0717:0	269.25	3189	3332	3461	3327	4.09

**Table 2:** Pinpointing the identity of PC 37:5 and PC O-38:5 in human plasma.

Molecular Ion	Calculated mass	PC 17:0/20:5	PC P-18:0/20:4	PC O-18:0/20:5	PC O-16:0/22:5	PE 18:0/22:5
TOF MS						
[M+H]^+^	794.5694/794.6058	794.5674	794.6037	794.6037	794.6037	794.5674
[M+CH_3_COO]^−^	852.5760/852.6124	852.5745	852.5745 ^b^	852.5745 ^b^	852.5745 ^b^	792.5408
[C_5_H_15_NO_4_P]+	184.0733	184.0731	184.0731	184.0731	184.0731	
[M+H−C_2_H_8_NO_4_P]+	653.5503					653.5414
MS/MS						
[C_17_H_33_O_2_]^−^	269.2486	269.2534				
[C_18_H_35_O_2_]^−^	283.2642					283.2689 ^a^
[C_20_H_29_O_2_]^−^	301.2173	301.2209		301.2209		
[C_20_H_31_O_2_]^−^	303.233		303.2373			
[C_22_H_33_O_2_]^−^	329.2486				329.2506	329.2546 ^a^

To confirm lipid identities in the potential presence of other isomeric and isobaric species we investigated the MS/MS spectrum of *m/z* 794.6 collected within the MS/MS^ALL^ analysis. As expected, the phosphoryl choline fragment ion at *m/z* 184.0731, derived from PC, was readily observed in the positive ion (Figure 6B). Interestingly, a fragment ion at *m/z* of 653.5548, corresponding to the neutral loss (NL) of *m/z* 141.0186, suggested that an overlapping phosphatidylethanolamine (PE) lipid was present. This species was tentatively identified as total PE 40:5, based on the characteristic NL [11] and the molecular ion. The elemental compositions and the theoretical masses of the protonated molecular ions of PE 40:5 and PC 37:5 are identical, however, in the negative ion mode these two lipids can be separated since PCs are primarily detected as acetate adducts, while PEs prefer a deprotonated form. Indeed, the predicted fragments supporting the identify of PE 40:5 and PC 37:5 and PC-O 38:5 were detected in corresponding MS/MS data in the negative ion mode derived from the MS/MS^ALL^ analysis. The MS/MS (*m/z* 792.5408) confirmed that PE 40:5 is present and predominantly in the form of the molecular species PE 18:0/22:5, confirmed by the detected acyl anions [21] (data not shown). In a similar way, the PC 37:5 (MS/MS of *m/z* 852.5745) can be confirmed, proposing the presence of the molecular species; PC 17:0/20:5, PC P-18:0/20:4, PC O-18:0/20:5 and PC O-16:0/22:5 (Figure 6C and Table 2). According to these results it can be expected that the content of isobaric and isomeric lipid entities in human plasma is very high. The complete cycle of MS and MS/MS from each polarity mode of each precursor mass, accompanied with high mass accuracy and selectivity, was found to be efficient in resolving interfering, isomeric and isobaric species in these types of complex samples. Thus, with the sequential precursor ion fragmentation technique, it can be expected that the lipidome coverage dramatically increases. Using the automated identification algorithms within lipidomics-driven lipid database searching tools [25] and starting from the most abundant signal, 404 molecular lipid species were tentatively identified in the plasma samples (Supplementary Table 1).

### 2.8. MS/MS^ALL^ is a Valid Methodology for the Assessment of Molecular Lipidomes

Assessing lipidomics investigations on human plasma has been a focus of recent studies [26]. Although significant analytical advances have been accomplished, there are still considerable discrepancies between reported lipid compositions. This has been observed in abundant lipid classes such as triacylglycerols (TAG), which are easy to detect. For instance, the TAG results reported by Graessler *et al.* [27], Oresic *et al*. [28] and Quehenberger *et al*. [6] differ not only in the number of TAG species, but also in their compositions. More importantly, to date, most studies have focused primarily on overall lipid compositions, with less attention given to the actual molecular species, bearing in mind their direct biological impact. We assumed that the acquired MS/MS^ALL^ data should have sufficient information to re-construct the molecular species lipid profile for plasma samples. As an example, the lipid profiles of PE and TAG were chosen for demonstration purposes. 

**Figure 7:**
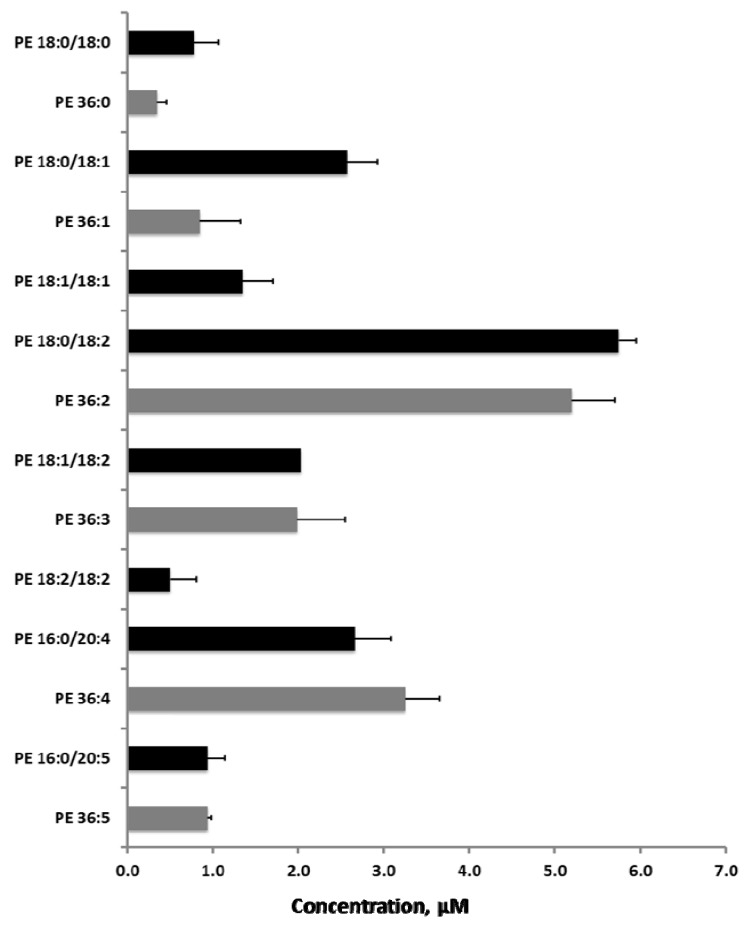
MS/MS^ALL^ delivers quantitative information on molecular PE species in human plasma. The obtained MS/MS information was utilized to identify the molecular PE species in human plasma. The identified molecular species were quantified by summing the peak intensities of the comprising acyl anions and normalized against the acyl anion signals of the pre-extraction spiked synthetic PE 17:0/17:0 standard and plasma volume (black bars), as described by Ejsing and colleagues [25]. For comparative analyses, the same samples were analyzed by conventional NLS of *m/z* 141.0 on a QqQ system (grey bars) as previously described [7]. The results of the PE 36:X series is shown. Collision energy was set to 50 eV for MS/MS^ALL^ and to 25 eV for NLS. Independent samples were analyzed and error bars indicate standard deviation (n = 5). Y-axis represents human plasma concentration in µM.

From the MS/MS^ALL^ analysis we were able to identify and quantify 33 distinct molecular PEs (data not shown). Since NL scanning on QqQ instruments has been broadly adopted to quantify PEs [11], as complete species, we wanted to explore how well the sequential precursor ion fragmentation results would match this type of analysis. We found that the concentrations of the molecular species and their corresponding total species agreed well with the QqQ results. The concentrations of the PE 36:X (X = 0–5) series and the corresponding molecular species is shown in Figure 7. For example, according to the QqQ analyses, human plasma contained approximately 3.2 µM PE 36:4. The corresponding molecular species PE 16:0/20:4 and PE 18:2/18:2 were determined by MS/MSALL to 2.7 and 0.5 µM respectively, which adds up to precisely the same amount as by the QqQ analysis. The identified lipid species and their concentrations compared well with data reported previously [13]. Similar observations were seen in the case of other lipid classes as well. For instance, MS/MS^ALL^ and QqQ analyses delivered closely similar concentrations of the ten most abundant molecular PC species (data not shown). However, slight discrepancies could be observed between the analyses. We reasoned that this mainly originated from improper isotopic correction and/or overlapping chemical background noise predominantly in the QqQ analysis. 

**Figure 8:**
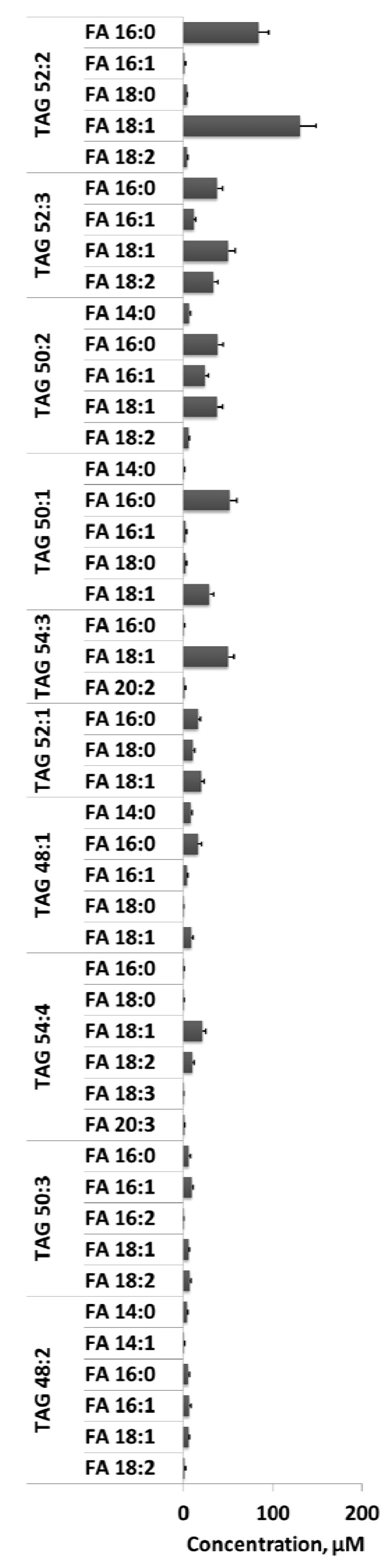
Quantification of the fatty acids making up complete TAGs in human plasma by MS/MS^ALL^. Positive ion mode MS/MS of TAGs generated diacyl fragment ions through neutral loss of fatty acids; these fragment ions were used to identify the molecular TAG species present [29]. The underlying fatty acid signals were quantified by the peak intensities of the diacyl fragment ions normalized against the diacyl fragment ion signal of the pre-extraction spiked synthetic TAG 17:0/17:0/17:0 standard and plasma volume. The concentrations of the ten most abundant TAG species are shown. Collision energy was set to 50 eV. Independent samples were analyzed and error bars indicate standard deviation (n = 17). Y-axis represents human plasma concentration in µM.

Similar results were obtained for the TAG lipid profile. 52 distinct complete species matched closely with other studies [12], however, the MS/MS^ALL^ acquisitions provided the underlying fatty acid compositions for each of the detected complete species. The ten most abundant endogenous TAGs and their fatty acid compositions are shown in Figure 8. Unfortunately, MS/MS^ALL^ experiments alone are insufficient to determine the quantity of each molecular TAG species, since the same fatty acids can be derived from multiple isomers/isobars. Additional specificity is needed to quantify TAGs, which can be achieved by LC separation or MS^3^ [29].

In summary, these results demonstrate that our methodology for sequential precursor ion fragmentation is fully capable of delineating molecular PE and TAG species. This capability does not apply to these lipid classes only, but it could be expanded to the whole lipidome. The agreement of our results with the lipid output from conventional QqQ approaches justifies the validity of MS/MS^ALL^. Thus, sequential the precursor ion fragmentation technique is not only rapid, but it also proves to be highly suitable for the characterization of lipidomes in most exhaustive detail with promising levels of quality, reproducibility and quantitation. 

## 3. Experimental

### 3.1. Materials

Methanol, acetic acid, 2,6-di-*tert*-butyl-4-methylphenol and ammonium acetate were from Sigma-Aldrich (St. Louis, MO, USA). Chloroform was from Rathburn Chemicals Ltd (Walkerburn, UK). Synthetic lipid standards were purchased from Avanti Polar Lipids Inc. (Alabaster, AL, USA), C/D/N Isotopes (Essex, UK), Matreya LLC (Pennsylvania, PA, USA) and Larodan Fine Chemical AB (Malmö, Sweden).

### 3.2. Sample Preparation and Extraction

Lipids were extracted from 10 μL of human fresh frozen plasma (FFP): extracted as described [23] in an automated fashion [7]. The antioxidant 2,6-di-*tert*-butyl-4-methylphenol was added as a sample protectant. Synthetic lipid standards were added to the samples prior to extraction. Their final concentrations in sample extracts were; 1.6 μM of LPC 17:0, PC 17:0/17:0, PA 17:0/17:0, PE 17:0/17:0, PG 17:0/17:0, PS 17:0/17:0, DAG 17:0/17:0, SM 18:1/12:0, TAG 17:0/17:0/17:0 and 3.2 μM of D6-CE 18:0, D6-FC and 0.125 μM of SPH 17:1 and 0.5 μM of S1P 17:1, CER 18:1/17:0, D3-GlcCER 18:1/16:0 and 0.25 μM of D3-LacCER 18:1/16:0, Gb3 18:1/17:0.

To investigate the dynamic quantification range, the samples were spiked with synthetic CER 17:1/18:0 at variable concentrations prior to extraction. The final concentrations in the sample extracts were; 5.0, 1.3, 0.313, 0.078, 0.020 and 0.005 µM. The sample set also consisted of appropriate sample blanks to ascertain and prevent any carryover. The final lipid extracts were dried under a gentle stream of nitrogen and reconstituted in chloroform:methanol (1:2, v/v) and stored under nitrogen at −20 °C prior to the lipidomic analyses.

### 3.3. MS and MS/MS Acquisitions on Hybrid Quadrupole Time-of-Flight

Plasma extracts were analyzed by flow injection analysis. Approximately 150 µL of a 5-fold diluted lipid extract in 5 mM ammonium acetate in chloroform:methanol (1:2, v/v) was delivered to the source by isocratic flow at 20 µL/min of methanol: isopropanol (3:1, v/v) with 5 mM ammonium acetate using a Shimadzu Prominence XR UFPLC autosampler and isocratic pump (Shimadzu Corporation, Kyoto, Japan). A second isocratic pump delivered a solution of 98% isopropanol and 2% methanol containing 5 mM ammonium acetate as a make-up flow to the source through a T-junction at a rate of 60 µL/min. Total flow was approximately 80 µL/min at point of entry into the DuoSpray® Source through the ESI probe. Source parameters included nebulizing gases GS1 at 20, GS2 at 15, curtain gas at 20, positive mode ion spray voltage at 5000, negative mode ion spray voltage at −4000, declustering potential at 40 V, and at an ESI source operating temperature of 300 °C. The atmospheric-pressure chemical ionization (APCI) probe and inlet were connected to a calibrant pump which delivers mass calibration solution (12 small molecule singly-charged compounds in both positive and negative modes for MS and MS/MS covering the *m/z* ranges acquired). Each sample was injected twice, to complete a positive mode and a negative mode experiment back-to-back upon polarity switching, according to the instrument manufacturer. An appropriate wash step and sample blanks were included to assess carryover. Positive and negative ion MS and sequential precursor ion fragmentation acquisitions were carried out on a TripleTOF™ 5600 System (AB SCIEX, Concord, ON) controlled with Analyst® TF 1.5.1 software with MS/MS^ALL^ mode activated to carry out series of product ion scans defined by the mass range and Q1 stepped masses as set by the user. An MS experiment was carried out from *m/z* 200–1,200 at an accumulation time of 300 ms, followed by 1,000 product ion experiments with 1,000 precursors evenly spaced from *m/z* 200.051 to *m/z* 1,200.051, measuring across *m/z* 100–1,500, accumulated for 300 ms each, collected in order from low to high *m/z*. Total time to carry out one MS/MS^ALL^ acquisition was 5.48 min. Collision energy for each MS/MS step was 50 ± 30 eV and −40 ± 30 eV respectively for positive and negative ion mode experiments.

### 3.4. Accurate MassData Processing

The acquired TOF MS and MS/MS^ALL^ data were processed with LipidView™ 1.1 software. The data supporting the quantitative aspect of the analysis, such as linearity and reproducibility were obtained using a target processing method that focused on exogenous lipid species in the fixed and serial-dilution internal standard solutions and based on the concentrations described above. To study the overall plasma lipidome composition identification of lipid species was performed in both positive and negative modes and the results combined after processing. The identification focused on the species with even carbon chains. The typical mass tolerance window for processing was set at 5 mDa and the peaks in MS/MS scans exceeding signal-to-noise of 3 were considered. To prevent false positive identifications within the dataset, the contribution of isotope peaks coming from lower mass species was removed within and between adjacent MS/MS scans using the information on accurate fragment elemental composition and elemental composition of the parent species [25]. The identified lipids were quantified against their corresponding internal standard, i.e. endogenous lipid against a corresponding internal standard of the same lipid class or closely related, in cases where appropriate internal standard is lacking, and normalized against sample volume. A total of 404 molecular lipid species were tentatively identified in the lipid data (Supplementary Table 1).

### 3.5. QqQ Shotgun Lipidomics Analyses

Shotgun lipidomics was performed on a QTRAP® 5500 instrumentation (AB SCIEX™, Concord, ON) equipped with a robotic nanoflow ion source (NanoMate HD, Advion Biosciences) as previously described [24]. Briefly, prior to sample introduction, the extracted plasma samples were diluted 5-fold in 5 mM ammonium acetate in chloroform:methanol (1:2, v/v). Operating gas pressure for the NanoMate HD was set to 0.7 psi and voltages were set to 1.4 kV for positive ion mode and −1.4 kV for negative ion mode, respectively. PIS and NLS were performed in both positive and negative ion modes as previously described [11,21]. The quadrupoles, Q1 and Q3, were operated at unit resolution and a scan rate of 200 Da/s was applied. The applied collision energy was optimized for each lipid class [7]. The instrument was operated using Analyst® 1.5 software. The acquired data was processed in LipidView™ software for lipid identification as described previously [25].

## 4. Conclusions

We demonstrated that the sequential precursor ion fragmentation (MS/MS^ALL^) technique successfully reveals the make-up of the human plasma lipidome. The method is justified by mirroring results obtained by conventional lipidomics methods. Orchestrated by accurate mass full scans and MS/MS information at each single mass unit from both polarity modes, MS/MS^ALL^ improves the precision of the lipid assessment and delivers quantitative information on the molecular lipid species, without hindering the discovery of unknowns. However, as MS/MS^ALL^ technique does not produce the complete picture of lipidomes; it requires supplementary techniques such as ion mobility [30] and OzID [20] to deliver the missing dimensions of the functional lipidome, as for instance the allocation of the position and the configuration of double bonds within the fatty acid moieties of lipid species and thereby disclose the detailed molecular lipid structures [31].

We also showed the capability of the sequential ion fragmentation technique to deliver quantitative information for more than 400 molecular lipid species in less than 12 min without compromising data quality; a feature that makes MS/MS^ALL^ an attractive set-up for high-throughput quantitative screenings [7]. In conjunction with this demonstrated high throughput, its ability to collect all accurate product ion data in an unbiased fashion paves the way to biomarker discovery, target discovery programs as it will prospectively shed new insights into the affected metabolic and signaling pathways, and the transition of lipidomics into clinical laboratories.

## Supplementary Files

Supplementary File 1 PDF-Document (PDF, 225 KB)
