# Supplementary material for: Shotgun Lipidomics by Sequential Precursor Ion Fragmentation on a Hybrid Quadrupole Time-of-Flight Mass Spectrometer
Source: Metabolites. 2012 Feb 20;2(1):195–213. doi: 10.3390/metabo2010195 (PMC3901199; doi:10.3390/metabo2010195)
Supplement: Supplementary File 1 — PDF-Document (PDF, 225 KB) [file metabolites-02-00195-s001.pdf]

Supplementary Table 1. Lipid species identified from both polarity modes in human plasma.

Lipid class	Lipid species
Cholesteryl Esters	CE 14:0
	CE 14:1
	CE 16:0
	CE 16:1
	CE 16:2
	CE 17:0
	CE 17:1
	CE 18:0
	CE 18:1
	CE 18:2
	CE 18:3
	CE 20:2
	CE 20:3
	CE 20:4
	CE 20:5
	CE 22:6
Diacylglycerols	DAG 32:0
	DAG 32:1
	DAG 34:0
	DAG 34:1
	DAG 34:2
	DAG 34:3
	DAG 36:1
	DAG 36:2
	DAG 36:3
	DAG 36:4
	DAG 36:5
	DAG 36:6
	DAG 38:4
	DAG 38:5
	DAG 38:6
	DAG 38:7
	DAG 38:8
	DAG 40:6
	DAG 40:7
	DAG 40:8
	DAG 42:6
	DAG 42:7
	DAG 44:8

Supplementary Table 1. *Cont.*

Lipid class	Lipid species
Monoacylglycerols	MAG 10:0
	MAG 12:0
	MAG 14:0
	MAG 14:1
	MAG 16:0
	MAG 16:1
	MAG 18:0
	MAG 18:1
	MAG 18:2
	MAG 18:3
	MAG 20:1
	MAG 20:2
	MAG 20:3
	MAG 20:5
	MAG 22:0
	MAG 22:1
	MAG 22:2
	MAG 24:1
Triacylglycerols	TAG 38:1
	TAG 40:0
	TAG 40:1
	TAG 44:0
	TAG 46:1
	TAG 46:2
	TAG 48:0
	TAG 48:1
	TAG 48:2
	TAG 50:0
	TAG 50:1
	TAG 50:2
	TAG 50:3
	TAG 50:4
	TAG 50:6
	TAG 50:8
	TAG 52:0
	TAG 52:1
	TAG 52:2
	TAG 52:3
	TAG 52:4
	TAG 52:5
	TAG 52:6
	TAG 54:1
	TAG 54:2
	TAG 54:3
	TAG 54:4

Supplementary Table 1. *Cont.*

Lipid class	Lipid species
Triacylglycerols	TAG 54:5
	TAG 54:6
	TAG 54:7
	TAG 56:10
	TAG 56:2
	TAG 56:3
	TAG 56:4
	TAG 56:5
	TAG 56:6
	TAG 56:7
	TAG 56:8
	TAG 56:9
	TAG 58:10
	TAG 58:11
	TAG 58:6
	TAG 58:7
	TAG 58:8
	TAG 58:9
	TAG 60:10
	TAG 60:11
	TAG 60:12
	TAG 60:9
	TAG 66:10
	TAG 66:9
	TAG 70:12
Free Fatty Acids	FFA 10:0
	FFA 12:0
	FFA 14:0
	FFA 14:1
	FFA 16:0
	FFA 16:1
	FFA 18:0
	FFA 18:1
	FFA 18:2
	FFA 18:3
	FFA 20:0
	FFA 20:1
	FFA 20:2
	FFA 20:3
	FFA 20:4
	FFA 20:5
	FFA 22:0
	FFA 22:1
	FFA 22:2

Supplementary Table 1. *Cont.*

Lipid class	Lipid species
Free Fatty Acids	FFA 22:4
	FFA 22:5
	FFA 22:6
	FFA 24:0
	FFA 24:1
Lysophosphatidic acids	LPA 12:0
	LPA 14:0
	LPA 16:0
	LPA 16:1
	LPA 18:2
	LPA 22:6
	LPA 24:0
Lysophosphatidylcholines	LPC 14:0
	LPC 16:0
	LPC 16:1
	LPC 18:0
	LPC 18:1
	LPC 18:2
	LPC 18:3
	LPC 20:3
	LPC 20:4
	LPC 20:5
	LPC 22:5
	LPC 22:6
Ether-linked/plasmalogen lysophosphatidylcholines	LPC O-16:0
	LPC O-18:0
	LPC O-18:1
	LPC O-18:2
Lysophosphatidylethanolamines	LPE 12:0
	LPE 14:0
	LPE 16:0
	LPE 18:0
	LPE 18:1
	LPE 18:2
	LPE 20:0
	LPE 20:3
	LPE 20:4
	LPE 20:5
	LPE 22:4
	LPE 22:6
Lysophosphatidylinositols	LPI 20:3
Phosphatidic acids	PA 28:0
	PA 28:1
	PA 30:1

Supplementary Table 1. *Cont.*

Lipid class	Lipid species
Phosphatidic acids	PA 32:0 (14:0/18:0, 16:0/16:0)
	PA 32:1
	PA 32:2
	PA 34:1
	PA 34:2
	PA 36:0 (18:0/18:0)
Phosphatidic acids	PA 36:1 (18:1/18:0)
	PA 36:2 (18:2/18:0, 18:1/18:1)
	PA 36:3 (18:0/18:3, 18:1/18:2)
	PA 36:6 (18:3/18:3)
	PA 38:3 (18:0/20:3)
	PA 38:4 (18:0/20:4, 18:1/20:3)
	PA 38:5 (18:1/20:4, 18:0/20:5)
	PA 38:6 (18:1/20:5)
	PA 40:4
	PA 40:5
	PA 40:6 (18:0/22:6, 18:1/22:5)
	PA 40:7 (18:1/22:6).
Ether-linked/plasmalogen phosphatidic acids	PA O-34:3
	PA O-36:1
	PA O-36:3
	PA-O 36:5
Phosphatidylcholines	PC 30:0 (16:0/14:0)
	PC 30:1
	PC 30:2
	PC 32:0 (14:0/O-18:0, 16:0/O-16:0)
	PC 32:1 (18:1/14:0, 16:1/16:0)
	PC 32:2 (16:1/16:1, 18:2/14:0)
	PC 34:0 (16:0/18:0)
	PC 34:1 (18:1/16:0, 16:1/18:0)
	PC 34:2 (16:0/18:2, 16:1/18:1)
	PC 34:3 (16:0/18:3, 16:1/18:2)
	PC 34:4 (20:4/14:0)
	PC 36:0 (18:0/18:0)
	PC 36:1(18:1/18:0, 16:0/20:1)
	PC 36:2 (18:1/18:1, 18:2/18:0, 16:0/20:2)
	PC 36:3 (16:0/20:3, 18:0/18:3, 18:1/18:2)
	PC 36:4 (16:0/20:4, 16:1/20:3, 18:2/18:2, 18:1/18:3)
	PC 36:5 (16:0/20:5)
	PC 36:6 (16:1/20:5)
	PC 38:0

Supplementary Table 1. *Cont.*

Lipid class	Lipid species
Phosphatidylcholines	PC 38:2 (18:0/ 20:2)
	PC 38:3 (18:0/ 20:3, 18:1/20:2)
	PC 38:4 (16:0/22:4, 18:0/20:4, 18:1/20:3)
	PC 38:5 (16:0/22:5, 18:0/ 20:5, 18:1/20:4, 18:2/20:3)
	PC 38:6 (16:0/22:6, 18:1/20:5, 18:2/20:4)
	PC 38:7 (16:1/22:6)
	PC 40:0
	PC 40:1
	PC 40:2
	PC 40:3
	PC 40:4 (18:0/22:4)
	PC 40:5 (18:0/22:5)
	PC 40:6 (18:0/22:6, 18:1/ 22:5)
	PC 40:7 (18:0/22:6)
	PC 40:8
	PC 42:0
	PC 44:1
	PC 44:2
	PC 44:3
	PC 44:4
Ether-linked/plasmalogen phosphatidylcholines	PC O-28:0 (10:0/O-18:0)
	PC O-32:0 (14:0/O-18:0)
	PC O-32:1 (14:1/O-18:0, 14:0/O-18:1)
	PC O-34:0 (16:0/O-18:0)
	PC O-34:1
	PC O-34:2 (16:2 / O-18:0)
	PC O-34:3
	PC O-34:4
	PC O-36:0 (18:0/O-18:0)
	PC O-36:1 (18:1/O-18:0)
	PC O-36:2 (18:2/O-18:0)
	PC O-36:3
	PC O-36:4
	PC O-36:5
	PC O-38:0 (20:0/O-18:0)
	PC O-38:1 (20:1/O-18:0)
	PC O-38:3 (20:3/O-18:0)
	PC O-38:4 (20:4/O-18:0)
	PC O-38:5 (20:5/O-18:0)
	PC O-38:6
	PC O-40:4 (22:4/O-18:0)

Supplementary Table 1. *Cont.*

Lipid class	Lipid species
Ether-linked/plasmalogen phosphatidylcholines	PC O-40:5
	PC O-40:6
	PC O-42:3
	PC O-42:5
	PC O-42:6
	PC O-44:5
Phosphatidylethanolamines	PE 32:0 (14:0/18:0 and 16:0./16:0)
	PE 34:0 (16:0/18:0)
	PE 34:1 (16:0/18:1, 18:0/16:1)
	PE 34:2 (16:0/18:2)
	PE 34:3 (16:0/18:3)
	PE 36:0 (18:0/18:0)
	PE 36:1 (18:0/18:1)
	PE 36:2 (18:0/18:2, 18:1/18:1)
	PE 36:3 (16:0/20:3, 18:1/18:2, 18:3/18:0)
	PE 36:4 (16:0/20:4, 18:2/18:2)
	PE 36:5 (16:0/20:5)
	PE 38:1
	PE 38:2
	PE 38:3 (18:0/20:3)
	PE 38:4 (18:0/20:4, 18:1/20:3, 16:0/22:4)
	PE 38:5 (16:0/22:5, 18:0/20:5, 18:1/20:4, 16:1/22:4)
	PE 38:6 (18:1/20:5, 16:0/22:6, 16:1/22:5, 18:2/20:4)
	PE 40:2
	PE 40:4 (18:0/22:4)
	PE 40:5(18:0/22:5)
	PE 40:6 (22:6/18:0)
	PE 40:7 (22:6/18:1)
	PE 42:5
	PE 42:6
	PE 42:7
	PE 44:6
	PE 46:0
	PE 46:1
	PE 48:2
Ether-linked/plasmalogen phosphatidylethanolamines	PE O-32:2
	PE O-36:1 (18:0/O-18:1)
	PE O-36:3 (18:2/O-16:1)
	PE O-36:6
	PE O-38:0
	PE O-38:5

Supplementary Table 1. *Cont.*

Lipid class	Lipid species
Ether-linked/plasmalogen phosphatidylethanolamines	PE O-38:6 (22:6/O-16:0)
	PE O-40:0
	PE O-40:1
	PE O-40:2
	PE O-40:5 (22:5/O-18:0)
	PE O-40:6 (22:6/O-18:0)
	PE O-42:2
	PE O-42:5
	PE O-44:0
	PE O-44:1
	PE O-44:4
	PE O-44:5
	PE O-46:2
	PE O-46:4
	PE O-46:5
	PE O-46:6
	PE O-48:2
	PE O-48:5
	PE O-48:6
Phosphatidylglycerols	PG 30:0
	PG 32:0
	PG 34:0 (16:0/18:0)
	PG 34:2 (16:0/18:2)
	PG 36:0 (18:0/18:0)
	PG 36:1 (18:1/18:0)
	PG 36:2 (18:0/18:2, 18:1/18:1)
	PG 36:4 (18:2/18:2)
	PG 36:6 (16:1/20:5)
	PG 38:5 (18:0/20:5)
	PG 38:6 (16:0/22:6)
	PG 40:6 (20:3)
	PG 42:10 (20:5/22:5)
	PG 42:9 (20:4/22:5)
Ether-linked/plasmalogen phosphatidylglycerols	PG O-36:0 (18:0/O-18:0)
	PG O-36:3 (18:3/O-16:0)
Phosphatidylinositols	PI 28:0
	PI 32:0 (14:0/18:0)
	PI 32:1 (14:0/18:1)
	PI 32:2 (16:1/16:1)
	PI 34:0 (16:0/18:0)
	PI 34:1 (16:1/18:0, 18:1/16:0)
	PI 34:2 (16:0/18:2)
	PI 36:0 (18:0/ 18:0)
	PI 36:1 (18:1/18:0)

Supplementary Table 1. *Cont.*

Lipid class	Lipid species
Phosphatidylinositols	PI 36:2 (18:1/18:1, 18:2/18:0)
	PI 36:3 (18:3/18:0, 18:1/18:2, 16:0/20:3)
	PI 36:4 (16:0/20:4, 18:2/18:2)
	PI 38:2 (18:0/ 20:2)
	PI 38:3 (18:0/20:3)
	PI 38:4 (18:0/20:4, 18:1/20:3)
	PI 38:5 (16:0/22:5, 18:0/20:5, 18:1/20:4)
	PI 38:6 (16:0/22:6)
	PI 40:5 (18:0/22:5)
	PI 40:6 (18:0/22:6)
Ether-linked/plasmalogen phosphatidylinositols	PI O-36:2 (18:2/O-18:0)
Phosphatidylserines	PS 34:0 (16:0/18:0)
	PS 34:3 (16:0/18:3)
	PS 34:7 (12:1/22:6)
	PS 36:0 (18:0/18:0)
	PS 36:1 (18:1/18:0)
	PS 36:2 (18:2/18:0, 18:1/18:1)
	PS 36:3 (18:1/18:2)
	PS 36:4 (18:2/18:2)
	PS 36:8 (14:2/22:6)
	PS 38:1 (18:0/20:1)
	PS 38:4 (18:0/20:4)
	PS 38:5 (20:4/18:1)
	PS 38:6 (18:2/20:4)
	PS 38:8 (16:2/22:6)
	PS 40:6 (18:0/22:6)
Ether-linked/plasmalogen phosphatidylserines	PS O-34:0 (16:0/O-18:0)
	PS O-40:7 (22:6/O-18:1)
Sphingomyelins	SM 30:1;2
	SM 30:2;2
	SM 30:3;4
	SM 32:2;3
	SM 34:0;2
	SM 34:1;2
	SM 34:1;3
	SM 34:2;2
	SM 36:0;4
	SM 36:1;2
	SM 36:2;2
	SM 36:2;3
	SM 36:3;2
	SM 36:3;3
	SM 38:0;2

Supplementary Table 1. *Cont.*

Lipid class	Lipid species
Sphingomyelins	SM 38:1;2
	SM 38:2;2
	SM 38:2;3
	SM 38:3;2
	SM 38:3;3
	SM 38:4;3
	SM 40:0;3
	SM 40:1;2
	SM 40:1;3
	SM 40:2;2
	SM 40:2;3
	SM 40:3;3
	SM 42:1;2
	SM 42:1;4
	SM 42:2;2
	SM 44:0;4
	SM 44:1;4
	SM 44:3:3
	SM 44:4;3
	SM 46:0;2
	SM 46:1;2
